# Characterization of Muscle Activation and Muscle Synergism in the ‘Forward Lunge’ Gait Movement of Badminton Players Using Surface Electromyography Sensors

**DOI:** 10.3390/s25061644

**Published:** 2025-03-07

**Authors:** Jian Jiang, Haojie Li, Chen Xiu

**Affiliations:** School of Athletic Performance, Shanghai University of Sport, Shanghai 200438, China; jiangjian@sus.edu.cn (J.J.); 202121070037@mail.bnu.edu.cn (H.L.)

**Keywords:** badminton, forward lunge, surface electromyography, muscle activation, muscle synergy, biomechanics

## Abstract

The ‘forward lunge’ is a crucial movement in badminton that demands effective muscle activation and coordination. This study compared the muscle activation patterns of professional and amateur male badminton players during this movement. A total of 24 players (12 professionals and 12 amateurs) participated, with surface electromyography (sEMG) used to measure the activity of 12 muscles on the right side during the lunge. The movement was divided into swing and support phases based on ground reaction force data. The sEMG signals were analyzed using integral EMG (iEMG) and root-mean-square (RMS) amplitude, and muscle synergy patterns were extracted via non-negative matrix factorization (NNMF) and k-means clustering. The results showed significantly higher iEMG and RMS values in muscles such as the gastrocnemius, biceps femoris, gluteus maximus, external oblique, and latissimus dorsi in professional players (*p* < 0.05), while no significant differences were observed in the tibialis anterior, vastus medialis, vastus lateralis, deltoideus, biceps, and soleus muscles. Muscle synergy analysis revealed three activation patterns in the professional group, compared to two in the amateur group. The additional synergy pattern in the professional players involved greater recruitment of lower limb and core muscles, especially during the support phase. In contrast, the amateur group showed earlier muscle activation but exhibited less efficient coordination. These findings suggest that muscle activation and coordination patterns in the forward lunge are influenced by playing level, highlighting the importance of lower limb and core training for badminton athletes to optimize performance and reduce injury risk.

## 1. Introduction

Badminton, as a highly technical and competitive sport, has been widely promoted and popularized worldwide in recent years [1]. The population of badminton participants is increasing, and the skill level of the participants is improving. However, despite the fact that badminton attracts a large number of beginners, it is still quite challenging for beginners to master the basic skills of badminton [2]. In particular, the learning and mastery of footwork is often the most difficult part of badminton for beginners when entering the field [3]. Badminton’s footwork movements include a variety of forms, such as forward and backward, side-to-side, and backward, which are designed to quickly and efficiently get players to the position where the badminton ball is located and ready to strike [4]. However, footwork in badminton is not just about moving quickly; it is also about optimizing the position of the shot and improving the efficiency and accuracy of the shot [5]. Of all the movements, footwork is considered the most fundamental and important part of badminton technique [6]. Studies have shown that good footwork not only helps to improve sports performance but also prevents sports injuries effectively because reasonable footwork can minimize unnecessary risks for athletes in the game [7].

In badminton, as a high-intensity and fast sport, the transient and explosive nature of its movements makes athletes susceptible to a variety of sports injuries during the game, especially those directly related to footwork movements. Previous studies have shown that ankle sprains, knee injuries, and muscle strains are the most common types of injuries in badminton [8]. The occurrence of these injuries is closely related to deficiencies in muscle activation patterns and imbalances in muscle control during the execution of stepping maneuvers by athletes. During lateral movement, athletes without proper muscle activation may be susceptible to overloading of the knee or spraining of the ankle [9]. Therefore, it is particularly important to study muscle activation patterns and intermuscular synergies. Muscle synergy not only provides the necessary support during high-intensity exercise but also maintains the stability and coordination of the body during exercise, thus reducing the occurrence of injuries [10]. For badminton players, optimizing muscle activation patterns, especially core and lower-limb muscles and their ability to work in synergy, is the key to preventing injury and improving performance.

‘Forward lunge’ is one of the most common and important forms of footwork in badminton, especially in the rapid attack and defense transition in the game [11]. In badminton, players often need to make a quick forward lunge and adjust their body position in mid-air or in contact with the ground in order to complete an accurate stroke [12]. Therefore, the forward lunge is considered one of the most indispensable skills for badminton players to move around the court and cope with the rapidly changing tempo of a match. However, as this maneuver requires athletes to exert a large amount of power and complete complex body transfers in a short period of time, beginners are particularly prone to knee, ankle, and lower back injuries due to improper posture or unskilled technique [13]. Therefore, the study of the muscle activation characteristics of this step and its muscle synergism is crucial for improving athletes’ skill levels and reducing the risk of injury. In addition, knowledge of how to effectively perform the ‘forward lunge’ will not only contribute to technical improvement but will also allow for rapid response and the optimization of court movement during the game.

Surface electromyography (sEMG) has become a popular tool for studying muscle activity in sports. By measuring the electrical signals of muscles on the surface of the skin, this technique is able to provide real-time data on the level of muscle activation, its timing, and its synergy [14]. Compared with traditional methods of motion analysis, sEMG technology has the advantages of being non-invasive, performed in real time, and highly accurate, and is widely used in sports science and biomechanics research [15,16]. In sports, the sEMG technique is used to analyze the activation patterns of the muscles of athletes during the execution of stride movements. For example, by evaluating metrics such as iEMG (integral electromyography) and the RMS (root mean square value) of the major muscle groups in different stride movements, researchers can analyze in detail the level of muscle engagement during the execution of a stride [17]. In addition, non-negative matrix factorization (NMF) techniques have been used to reveal the synergies between muscle groups [18], which is important for understanding how muscle groups work together and respond synergistically to dynamic changes in the stepping action. Through these techniques, insights into muscle control and movement efficiency can be provided to badminton players in order to optimize training methods and reduce the occurrence of injuries.

The aim of this study was to systematically analyze the muscle activation patterns and muscle synergies of badminton players during the execution of the ‘forward lunge’ step using surface electromyography (EMG). Although the application of EMG technology in other sports has made significant progress, there is still a lack of research in the specific sport of badminton. By carefully analyzing the muscle activation characteristics of badminton footwork, this study can not only provide a new scientific basis for improving badminton players’ footwork technique but also provide more specific guidance for athletes’ body control and sports injury prevention during competition. The innovation of this study is the incorporation of surface EMG sensors to analyze the complexity of muscle synergies in a quantitative manner. In addition, this study will explore the potential effects of different training methods on muscle activation patterns and sports performance, providing a new theoretical basis for the specialized training of badminton players. Therefore, the research results will have an important role in promoting academic research in the fields of sports biomechanics, badminton training, and sports medicine, as well as practical significance for the improvement of footwork techniques and injury prevention in actual sports training.

In recent years, there has also been some evidence from studies on muscle activation in badminton players, particularly involving muscle coordination and injury prevention at different levels of exercise. Lin [19] analyzed the muscle activation patterns of badminton players when performing fast change in direction movements using surface EMG techniques and found that advanced players had more efficient muscle coordination, and the activation of the core muscles was significantly superior to beginners. Similarly, Cheng [20] showed that the synergistic action of the quadriceps, gastrocnemius, and gluteus maximus muscles is essential for reducing knee joint load and injury risk during stride movements in badminton players. These studies provide a theoretical basis for the present study and further suggest that it is important to improve performance and reduce sports injuries by thoroughly investigating muscle activation patterns and their synergistic effects in athletes of different levels.

A review of the literature shows that although studies have revealed differences in gait and muscle coordination among athletes, there is still a lack of quantitative analyses of muscle activation and synergistic patterns specific to the badminton ‘forward stride’ movement. This study aims to fill this gap and to investigate the application of electromyography in badminton techniques and training in order to provide more specific guidelines for badminton players during training and injury prevention.

The main contribution of this study is to quantitatively analyze the muscle activation and coordination patterns of badminton players during the ‘forward stride’ movement by means of surface electromyography (sEMG), revealing significant differences in muscle coordination and activation patterns between professional and amateur players. In addition, this study investigated the effects of different training levels on muscle activation patterns, highlighting the importance of lower limb and core training to improve performance and reduce injury risk. The results of this study provide a new theoretical basis and practical guidance for badminton training and sports injury protection and have important academic and applied value.

## 2. Participants and Methods

### 2.1. Participants

Twenty-four male badminton players were selected as participants in this study, including 12 badminton players at national level 2 and above (professional group) and 12 badminton enthusiasts with at least 1 year of badminton training experience (amateur group). All participants were right-sided dominant and signed an informed consent form before the start of the experiment. In order to ensure the accuracy and consistency of the experimental data, the selection and exclusion criteria of participants in this study were strictly defined to ensure that all the data could reflect the real situation of muscle activation and synergy.

Inclusion Criteria:

Gender and age: all participants were male and aged between 18 and 30 years.

Badminton sports background:

Professional group: participants were national level 2 and above badminton players; national level 2 players were in the top three players in professional badminton provincial competitions, had at least 6 or more years of badminton training experience, and had participated in at least one provincial badminton competition.

Amateur: participants were amateur athletes with at least 1 year of badminton training experience and regularly participated in badminton training but had not participated in professional badminton competitions.

Dominant hand type: all participants were right-handed dominant, i.e., they were accustomed to using their right hand for most of their activities in daily life.

Health status: all participants passed health screening prior to the experiment to confirm that they were free of cardiovascular disease, respiratory disease, or other health problems that would interfere with exercise capacity.

Exclusion criteria:

To ensure the accuracy and consistency of the study data, participants with the following conditions were excluded:

Lower-extremity injuries: participants were excluded if they had (within the past 3 months) lower extremity muscle or joint injuries, including but not limited to muscle strains, ligament injuries, and joint sprains.

Neurological or chronic disease: participants with a history of any neurological or musculoskeletal disease or chronic disease (e.g., arthritis, diabetes, etc.) were excluded.

Medication: Participants who were on medication or used medication that could affect muscle function were excluded.

Inability to complete the experimental task: participants were also excluded if they were unable to complete the experimental task due to health problems or other reasons.

Training frequency and duration:

Professional group: These athletes train at least 5 times per week for 2 to 3 h per training session, which consist of badminton technique training, physical training, tactical drills, and match simulation. The training focuses on the refinement of techniques and the ability to adapt to the game.

Amateur Group: Players in the amateur group train twice a week, and each training session lasts for 1 to 2 h. The training content mainly focuses on the improvement of basic badminton skills, such as pace practice, serving and receiving, etc., and these athletes do not undergo high-intensity physical training.

Sports injuries and medical history:

All participants underwent a detailed health screening prior to the experiment to confirm that there was no history of severe trauma to the lower limbs or diseases affecting sports performance. The screening included but was not limited to, joint range of motion testing, muscle strength testing, and inquiries about past sports injuries. Participants were excluded if they had a history of severe lower-extremity trauma (e.g., fractures of the knee or hip, ligament injuries, etc.) from which they had not fully recovered.

Gender selection and data standardization:

Although there are differences in physiological structure and athletic performance between men and women, the basic technical movements of badminton (e.g., the ‘forward stride’ stride) have a high degree of similarity between genders. Therefore, only male athletes were selected for this study to ensure the uniformity of results and comparability of data. In addition, in order to exclude the influence of gender differences on the results, all data in this study were standardized during data analysis, thus ensuring comparability across groups.

Basic information and statistical analysis:

Before the start of the study, the basic information of all participants (e.g., age, height, weight, exercise experience, etc.) was statistically analyzed to ensure that there were no significant differences in basic characteristics between the two groups (see Table 1). The fact that there were three overweight people with high BMI in the professional group and four overweight people in the amateur group did not affect the validity of the experiment; this is because we would have used standardized methods for all data when counting in order to eliminate the potential influence of inter-individual differences on the results.

### 2.2. Data Acquisition

**Figure 1:**
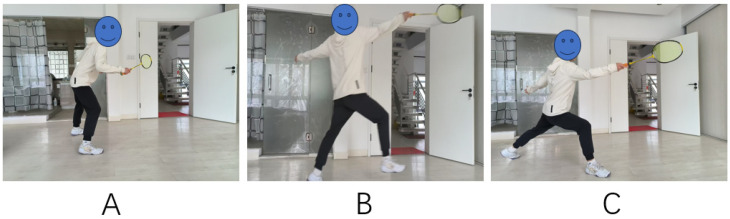
(**A**) The start phase; (**B**) the step-up phase, and (**C**) the end phase.

In this study, the badminton ‘forward lunge’ is a left–right symmetrical movement, so we chose the participant’s dominant right side for the study. As the movement form and muscle activation pattern of this step is similar on the left and right sides, focusing on the right side of the ‘forward lunge’ effectively represents the muscle activation characteristics of this movement [21]. In addition, all participants’ dominant side was the right side, and this standardization helped to reduce the effect of individual differences. The ‘forward lunge’ is divided into three phases: the start phase, the step-up phase, and the end phase (Figure 1). The appearance of the front lunge of a professional badminton player is characterized by precise pacing, smooth movement, and body stability. The knee flexion angle of the front leg is moderate (close to 90 degrees), the torso remains upright, and the foot rolls naturally from the heel to the toe when landing, so the overall movement is coordinated and efficient. In contrast, the forward lunge of the amateur athlete may appear to be unsteady, stiff, and wobbly. The knee flexion angle of the front leg may be too large or too small; the torso tends to lean forward or backward; the foot landing is not natural; and the overall movement does not appear to be coherent and coordinated. Figure 1 shows the correct technical movement.

**Figure 2:**
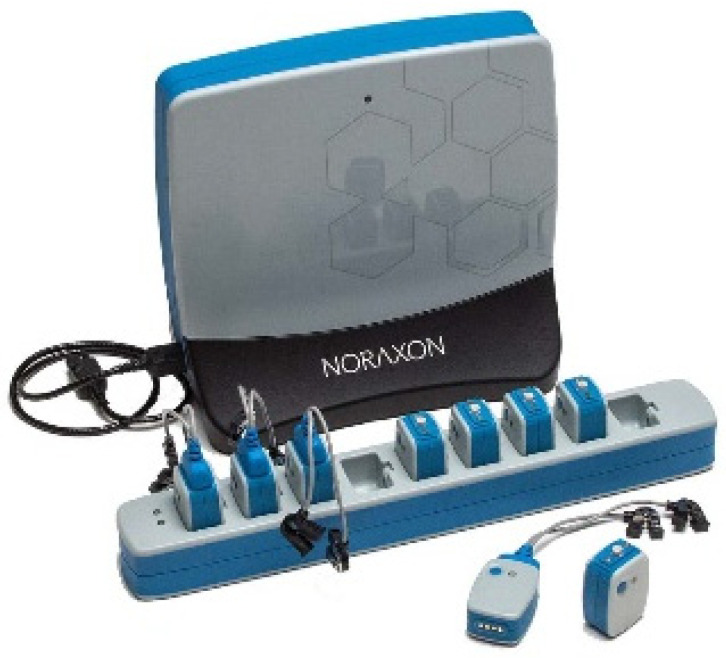
EMG sensors.

In this study, a Noraxon wireless surface EMG system (Noraxon, Ultium EMG, Scottsdale, Arizona, USA) (2000 Hz) (Figure 2) was used to collect surface EMG data from 12 muscles of the right limb: OE (external oblique), LD (latissimus dorsi), DD (deltoideus), BP (biceps), BD (brachioradialis), Gmax (gluteus maximus), VM (vastus medialis), VL (vastus lateralis), BF (biceps femoris), TA (tibialis anterior), GAS (gastrocnemius), and SOL (soleus) muscles.

To ensure the reliability and consistency of the data, each participant performed the forward lunge movement five times, and surface EMG data were recorded for each repetition. The experimental data used for analysis were the average values of these five trials, which helped to minimize the impact of potential outliers and variability in individual trials. In order to simplify the experimental process and increase the consistency of data collection, the EMG sensor was used to collect data only on the right side of the body.

### 2.3. Division of the ‘Forward Lunge’ Step Cycle

Ground reaction force data were processed using Visual 3D software V6 to delineate the walking cycle. The first frame with a vertical ground reaction force (vGRF) <10 N was defined as the point of departure, and the electromyographic data between the time the right foot left the ground and the time that the lateral foot left the ground again was intercepted and identified as a complete ‘forward lunge’ walking movement. The data were divided into two phases considering where the right foot followed the ground, the right lateral swing phase, and the right lateral support phase.

### 2.4. EMG Data Processing

The intercepted EMG data were preprocessed using Matlab R2022a, which included mean removal, bandpass filtering (4th order Butterworth filter, 10–400 Hz), full-wave rectification, and smoothing using a low-pass filter. The low-pass filter applied for smoothing was a 4th-order Butterworth filter with a cut-off frequency of 20 Hz. This filter was designed to effectively remove high-frequency noise while preserving the relevant muscle signal characteristics. The 4th-order Butterworth filter was chosen to ensure a flat passband and a smooth roll-off at the cut-off frequency.

Following this, the root-mean-square amplitude was calculated, and the signals were normalized both in terms of time and amplitude. At this stage, each subject’s EMG signal was represented as the original matrix V, which was then discretized for further analysis.

Integral EMG refers to the area under the EMG curve and the coordinate axis. This metric reflects the number of motor units recruited and discharged during muscle activity per unit of time, as well as the number of muscle fibers involved in the movement [22]. Integral EMG provides an intuitive representation of the muscle’s force generation capacity, with the unit of measurement being μV-s. The formula for integral EMG is as follows:

$$\begin{array}{c} iEMG=\frac{1}{T\int_{0}^{T}\left|X\left(t\right)\right|dt} \end{array}$$

Root mean square amplitude (RMS) is a commonly used feature in time domain analysis and is widely used for the feature extraction of EMG signals. MS is used as a measure of the magnitude of an EMG signal and can reflect the strength of muscle activation. During muscle activity, a higher RMS value usually implies stronger muscle contraction. MS calculates the average energy of the signal, especially when analyzing muscle fatigue, and the RMS value sometimes decreases over time, indicating a lower level of muscle activation [23]. Here, Xi denotes each data point in the time domain signal, and N is the total number of data points of the signal. With this formula, RMS is able to quantify the amplitude of the signal, which, in turn, reflects muscle activation [24].

$$\mathrm{RMS}=\sqrt{\frac{1}{N}\overset{N}{\underset{i=1}{\sum }}x_{i}^{2}}$$

### 2.5. Extraction of Muscle Synergies

The non-negative matrix factorization (NNMF) algorithm was applied to the EMG data matrix to extract muscle synergy. The algorithm assumes that the EMG envelope from each muscle can be described as a set of linear combinations of muscle synergy vectors (W) activated by activation coefficients (H) [25]. This assumption can be described as follows:

$$V_{m\times t}=W_{m\times n}\times H_{n\times t}+E$$

where m is 12 muscles, t is 101 data points after normalization, and n is the number of muscle synergies. n is the residual matrix. n is taken in turn from 1 to 12, and for each integer n, the original matrix V is subjected to NNMF analysis until the residuals E of the reconstructed matrix Vr and the original matrix V are iterated to a minimum. In this process, the final W and H are obtained for the current number n. Normalization with the maximum value of W ensures that all values in W are between 0 and 1.

The variance accounted for (VAF) is commonly used to test the degree of reconstruction in the reconstructed matrix Vr with respect to the original matrix V and to determine the number of synergies n. The value of this parameter ranges from 0 to 1, with larger values indicating higher reconstruction accuracy. The formula for VAF is as follows:

$$\mathrm{VAF}=1-\frac{{\left(V-\mathrm{Vr}\right)}^{2}}{V^{2}}$$

where V is the original matrix, and Vr is the reconstructed matrix, i.e., W*H.

The most commonly used criterion is VAF > 0.9, so in this study, we chose the n value of VAF > 0.9 as the number of muscle synergies under a specific movement for each subject, and the currents of W and H were used as the final synergy outputs [26].

To evaluate the quality of the muscle synergy models, two commonly used metrics are the variance accounted for (VAF) and the coefficient of determination (R-squared). VAF measures the proportion of variance in the data explained by the model, with a threshold of VAF > 0.9 often considered indicative of a well-fitted model [27]. R-squared, on the other hand, quantifies the goodness-of-fit by assessing the correlation between the predicted and actual values, with higher values indicating better model performance [28]. “For instance, in the study by Kim et al. [29], both VAF and R-squared were employed to assess the quality of muscle synergy extraction. The authors reported a VAF value of 0.92, indicating that 92% of the variance in muscle activity was captured by the model, alongside an R-squared value of 0.88, demonstrating a strong correlation between the model’s predictions and the observed data”.

In order to reveal the specific synergy patterns of the badminton ‘forward lunge’, the synergy patterns of each group for the footwork movements were clustered using k-means cluster analysis to form reference synergy patterns. The number of clusters, k, was determined using the elbow method, which is a common technique for selecting the optimal value of k in clustering algorithms. The elbow method works by plotting the within-cluster sum of squares (WCSS), also known as inertia, for various values of k. WCSS measures the total squared distance between each data point and its cluster centroid, indicating how tightly the points are grouped within each cluster. We used k-means clustering to group muscle synergies. The optimal number of clusters (k) was determined using the elbow method, which evaluates the within-cluster sum of squares (WCSS) for different values of k. Specifically, we calculated the WCSS for k ranging from 1 to 10 and identified the optimal k as the point where the rate of decrease in WCSS significantly slowed down, forming an ’elbow’ shape. This method ensures a balance between model complexity and the ability to explain the variance in the data. Based on this analysis, we selected k = [optimal k] as the optimal number of clusters for our dataset [30].

As k increases, WCSS typically decreases because data points are assigned to more clusters, reducing the distance between points and their centroids. The key idea of the elbow method is to identify the point where the rate of decrease in WCSS slows down significantly, forming an ‘elbow’ shape in the plot. This point suggests that adding more clusters beyond this number does not substantially improve the clustering. In this study, k = 3 was selected based on the elbow method, as this value provided a good balance between capturing the diversity of the synergy patterns and avoiding overfitting by creating too many clusters.

Then, the Pearson correlation coefficient (r) was used to match the synergy patterns of individual subjects with the reference synergy patterns of the group. When the correlation coefficient (r) exceeded 0.6, the synergy patterns were considered similar and grouped into the same category. After completing the matching of the W matrix (muscle activation time series), the corresponding H matrix (muscle contribution weights) of the original matched pair was also automatically classified into the same category.

### 2.6. Statistical Analyses

Descriptive statistics were performed on basic information and EMG data, and the results were expressed as the mean ± standard deviation (Mean ± SD). Comparisons between the two groups were made using the independent samples *t*-test. The *t*-test was used to compare the differences between the two groups and, thus, to test the hypothesis of differences in muscle activation between professional and amateur badminton players, with a *p*-value of <0.05 indicating a significant difference between the two groups. In addition, muscle synergy data were analyzed using weights and coefficient characteristics.

## 3. Results

### 3.1. iEMG and RMS Results

As shown in Table 2, the professional group was significantly higher (*p* < 0.05) than the amateur group for the integral EMG values of GAS, BF, Gmax, OE, and LD, whereas no statistically significant differences were observed in TA, VM, VL, DD, BP, B and SOL muscles (*p* > 0.05).

According to Table 3, the root-mean-square amplitude (RMS) of the professional group was significantly greater than that of the amateur group in GAS, VM, Gmax, OE, and LD (*p* < 0.01). However, the differences in root mean square amplitudes for TA, VL, BF, SOL, DD, BP, and BD did not reach statistical significance (*p* > 0.05). This suggests that there were fewer differences between the professional and amateur groups in these muscle groups.

### 3.2. Muscle Synergy Results

**Figure 3:**
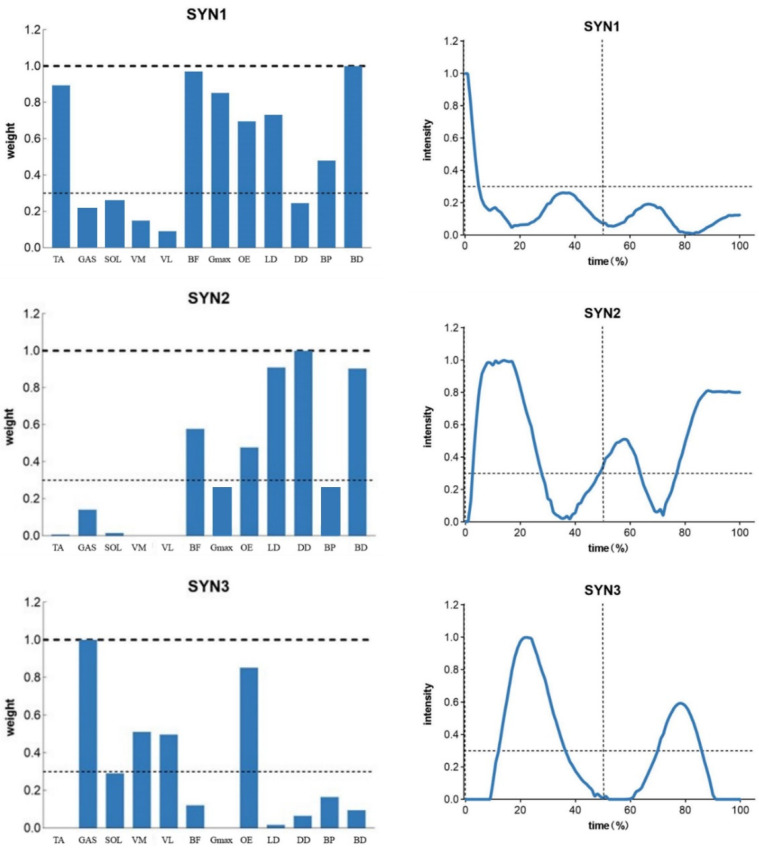
Muscle synergistic pattern of ‘forward lunge’ in the professional group.

**Figure 4:**
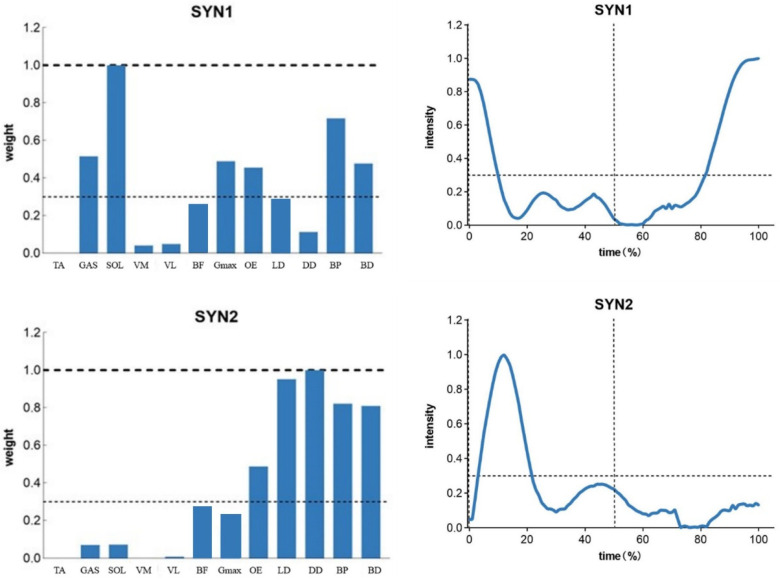
Muscle synergistic patterns of the ‘forward lunge’ step movement in the amateur group.

Activation above 0.3 indicates muscle activation in the pattern. Figure 3 shows the activation patterns and activation curves of the professional group in the ‘forward lunge’ stepping movement; the professional group had three activation patterns in the stepping movement, while the amateur group had two activation patterns (Figure 4), and more muscles were involved in the first two activation patterns of the professional group. Muscle groups were involved in the stepping movement, and the activation curves indicated that the activation time in the professional group was during the stepping support, whereas the amateur group performed the activation at the beginning of the stepping movement.

In addition, the professional group had one more new activation pattern, in which mainly the GAS, SOL, VM, VL, and OE lower limb and core muscle groups participated in the activation pattern.

## 4. Discussion

As shown in Table 2 and Table 3, the professional group exhibited significantly higher integral EMG (iEMG) and RMS values in muscles such as the gastrocnemius (GAS), gluteus maximus (Gmax), external oblique (OE), and latissimus dorsi (LD) compared to the amateur group (*p* < 0.05). Specifically, GAS, which plays a key role in plantar flexion and propulsion, showed a significantly higher iEMG value of 0.290 ± 0.079 in the professional group versus 0.167 ± 0.014 in the amateur group (*p* = 0.004). Additionally, RMS values for GAS were also significantly higher in the professional group (0.402 ± 0.066 vs. 0.312 ± 0.021, *p* = 0.002). These findings suggest that professional players engage the gastrocnemius muscle more intensely during the forward lunge, likely due to the greater demand for explosive lower limb power and faster movements. The higher activation of GAS in the professional group emphasizes the critical role of calf strength in generating force and facilitating rapid, controlled movements [10]. In parallel, the Gmax muscle, which is vital for hip extension, stability, and power during the lunge, showed significantly higher iEMG and RMS values in the professional group (iEMG: 0.273 ± 0.064 vs. 0.197 ± 0.050, *p* = 0.029; RMS: 0.345 ± 0.042 vs. 0.267 ± 0.022, *p* = 0.003). The increased activation of Gmax in professionals highlights their superior ability to generate lower body force, contributing to their more effective movement and power output. This is consistent with previous studies, which suggest that strong gluteal muscle activation is essential for dynamic movements like lunging, which require quick acceleration and deceleration [31]. Furthermore, the OE and LD muscles, which are involved in core stability and upper body force transfer, also demonstrated significantly higher iEMG and RMS values in the professional group. For OE, the iEMG value was 0.864 ± 0.043 in professionals versus 0.830 ± 0.022 in amateurs (*p* = 0.016), and the RMS value was 0.869 ± 0.041 vs. 0.839 ± 0.021 (*p* = 0.007). Similarly, the LD muscle exhibited higher activation in the professional group (iEMG: 0.347 ± 0.038 vs. 0.253 ± 0.048, *p* = 0.007; RMS: 0.413 ± 0.039 vs. 0.370 ± 0.032, *p* = 0.004). These muscles, integral to maintaining stability and supporting force transmission from the lower to the upper body, were activated to a greater extent in skilled players, reflecting the importance of core and upper body coordination in maintaining balance and optimizing performance during complex, dynamic movements such as the forward lunge. The enhanced activation of these muscles in professionals underscores their ability to transfer power more efficiently and maintain better control over their movements during high-intensity, quick transitions [32]. Conversely, there were no significant differences between the professional and amateur groups in the activation of muscles such as the tibialis anterior (TA), vastus medialis (VM), vastus lateralis (VL), soleus (SOL), deltoideus (DD), biceps (BP), and brachioradialis (BD) (*p* > 0.05). These results suggest that although these muscles are certainly involved in the forward lunge, they may not exhibit the same degree of differentiation between the two groups in terms of activation levels. For example, the TA muscle, which is crucial for dorsiflexion and the stability of the ankle, did not show significant differences in either iEMG (*p* = 0.642) or RMS (*p* = 0.237), with values of 0.384 ± 0.127 vs. 0.441 ± 0.129 and 0.459 ± 0.075 vs. 0.495 ± 0.091, respectively. This might indicate that both professional and amateur players rely on similar levels of TA activation for basic stability during the forward lunge, with differences in the lower limb primarily affecting more powerful and large muscles like the GAS and Gmax [33].

Muscle synergy analysis revealed a clear distinction in the number of synergies utilized by the two groups. The professional group demonstrated three activation patterns, including one additional synergy that involved GAS, SOL, VM, VL, and OE muscles. This additional synergy likely reflects the professional players’ ability to better coordinate lower limb and core muscles during the support phase of the lunge [34]. In comparison, the amateur group relied on only two synergies, with earlier muscle activation at the beginning of the movement but the limited engagement of critical muscle groups during the support phase. These findings suggest that professional players possess a more refined neuromuscular strategy that enables them to perform complex movements with greater precision, stability, and energy efficiency [35].

The differences observed in muscle activation and synergy patterns between the two groups highlight the importance of targeted training to improve performance and reduce injury risk [36]. Professional players’ greater activation of lower limb and core muscles during the support phase suggests that these muscle groups play a vital role in stabilizing the body and generating explosive force during badminton-specific movements [19]. Training programs for amateur players should, therefore, emphasize strengthening the GAS, Gmax, VM, VL, and OE muscles, as well as improving coordination and synergy among these muscle groups. Incorporating neuromuscular training, plyometric exercises, and core stabilization drills may help bridge the gap in movement efficiency and control [37].

Moreover, the reduced muscle activation and limited synergies in amateur players may increase their susceptibility to injuries, particularly in the knee and ankle joints [38]. The improper engagement of stabilizing muscles like Gmax and SOL during high-impact movements could lead to joint instability and overuse injuries [20]. Injury prevention programs should focus on improving dynamic stability, proprioception, and postural control to enhance movement quality and reduce injury risks [39].

While this study provides valuable insights into the biomechanics of the ’forward lunge’ in badminton, certain limitations should be addressed. First, this study focused solely on male participants, and future research should explore potential gender differences in muscle activation and synergy patterns. Second, the sample size was relatively small, and expanding the participant pool may yield more generalizable results. Additionally, the effects of fatigue, match intensity, and long-term training adaptations were not considered in this study, which could influence muscle activation and coordination strategies. Future studies should incorporate these factors to provide a more comprehensive understanding of badminton biomechanics.

## 5. Conclusions

This study demonstrates that professional badminton players exhibit more efficient muscle activation and coordination during the ’forward lunge’ compared to amateur players. Specifically, professionals showed higher iEMG and RMS values in key muscles, such as GAS, Gmax, OE, and LD, indicating stronger activation and motor unit recruitment. Additionally, muscle synergy analysis revealed that professionals use an extra activation pattern involving GAS, SOL, VM, VL, and OE muscles, highlighting greater engagement of the lower limb and core muscles during the support phase. These findings underscore the importance of lower limb and core training for improving performance and injury prevention in badminton. Future studies could explore gender differences, fatigue effects, and injury mechanisms for a more comprehensive understanding of badminton biomechanics.

## Figures and Tables

**Table 1:** Basic information on participants.

Indicator	Professional Group	Amateur Group	*p*-Value	FDR-Adjusted *p*-Value
Age (year)	25.3 ± 3.2	24.8 ± 2.7	0.678	0.678
Height (cm)	177.5 ± 5.4	176.3 ± 4.8	0.523	0.704
Weight (kg)	73.2 ± 7.4	71.8 ± 6.9	0.564	0.704
BMI (kg/m^2^)	23.2 ± 2.1	22.9 ± 2.3	0.704	0.812

**Table 2:** Comparison of integral EMG data for the ‘forward lunge’ step maneuver (Mean ± SD).

Muscle	Professional Group	Amateur Group	*p*-Value	FDR-Adjusted *p*-Value
TA	0.384 ± 0.127	0.441 ± 0.129	0.642	0.963
GAS	0.290 ± 0.079	0.167 ± 0.014	0.004	0.024
VM	0.230 ± 0.030	0.262 ± 0.042	0.290	0.580
VL	0.606 ± 0.020	0.548 ± 0.141	0.399	0.684
BF	0.213 ± 0.041	0.150 ± 0.016	0.028	0.048
Gmax	0.273 ± 0.064	0.197 ± 0.050	0.029	0.047
OE	0.864 ± 0.043	0.830 ± 0.022	0.016	0.036
LD	0.347 ± 0.038	0.253 ± 0.048	0.007	0.008
SOL	0.686 ± 0.251	0.732 ± 0.231	0.732	0.976
DD	0.437 ± 0.063	0.443 ± 0.055	0.844	0.731
BP	0.483 ± 0.082	0.482 ± 0.195	0.973	0.973
BD	0.789 ± 0.059	0.791 ± 0.053	0.884	0.864

Note. OE (external oblique), LD (latissimus dorsi), DD (deltoideus), BP (biceps), BD (brachioradialis), Gmax (gluteus maximus), VM (vastus medialis), VL (vastus lateralis), BF (biceps femoris), TA (tibialis anterior), GAS (gastrocnemius), and SOL (soleus) muscles.

**Table 3:** Comparison of RMS data (Mean ± SD) for the ‘forward lunge’ step maneuver (Mean ± SD).

Muscle	Professional Group	Amateur Group	*p*-Value	FDR-Adjusted *p*-Value
TA	0.459 ± 0.075	0.495 ± 0.091	0.237	0.406
GAS	0.402 ± 0.066	0.312 ± 0.021	0.002	0.012
VM	0.392 ± 0.027	0.308 ± 0.035	0.004	0.012
VL	0.626 ± 0.011	0.595 ± 0.121	0.384	0.517
BF	0.296 ± 0.041	0.276 ± 0.012	0.118	0.236
Gmax	0.345 ± 0.042	0.267 ± 0.022	0.003	0.012
OE	0.869 ± 0.041	0.839 ± 0.021	0.007	0.017
LD	0.413 ± 0.039	0.370 ± 0.032	0.004	0.012
SOL	0.704 ± 0.135	0.761 ± 0.102	0.388	0.517
DD	0.507 ± 0.066	0.503 ± 0.046	0.851	0.851
BP	0.517 ± 0.061	0.526 ± 0.064	0.755	0.824
BD	0.701 ± 0.050	0.691 ± 0.064	0.722	0.824

## Data Availability

Data included in the article.
